# Evaluation of the systematic error in using 3D dose calculation in scanning beam proton therapy for lung cancer

**DOI:** 10.1120/jacmp.v15i5.4810

**Published:** 2014-09-08

**Authors:** Heng Li, Wei Liu, Peter Park, Jason Matney, Zhongxing Liao, Joe Chang, Xiaodong Zhang, Yupeng Li, Ronald X Zhu

**Affiliations:** ^1^ Department of Radiation Physics UT MD Anderson Cancer Center Houston TX; ^2^ Department of Radiation Oncology Mayo Clinic Phoenix AZ; ^3^ Department of Radiation Oncology Emory University Atlanta GA; ^4^ Department of Radiation Oncology University of North Carolina Chapel Hill NC; ^5^ Department of Radiation Oncology UT MD Anderson Cancer Center Houston TX; ^6^ Applied Research Varian Medical Systems Palo Alto CA USA

**Keywords:** 4D CT, motion management, dose calculation, proton therapy, pencil beam scanning

## Abstract

The objective of this study was to evaluate and understand the systematic error between the planned three‐dimensional (3D) dose and the delivered dose to patient in scanning beam proton therapy for lung tumors. Single‐field and multifield optimized scanning beam proton therapy plans were generated for ten patients with stage II‐III lung cancer with a mix of tumor motion and size. 3D doses in CT datasets for different respiratory phases and the time‐weighted average CT, as well as the four‐dimensional (4D) doses were computed for both plans. The 3D and 4D dose differences for the targets and different organs at risk were compared using dose‐volume histogram (DVH) and voxel‐based techniques, and correlated with the extent of tumor motion. The gross tumor volume (GTV) dose was maintained in all 3D and 4D doses, using the internal GTV override technique. The DVH and voxel‐based techniques are highly correlated. The mean dose error and the standard deviation of dose error for all target volumes were both less than 1.5% for all but one patient. However, the point dose difference between the 3D and 4D doses was up to 6% for the GTV and greater than 10% for the clinical and planning target volumes. Changes in the 4D and 3D doses were not correlated with tumor motion. The planning technique (single‐field or multifield optimized) did not affect the observed systematic error. In conclusion, the dose error in 3D dose calculation varies from patient to patient and does not correlate with lung tumor motion. Therefore, patient‐specific evaluation of the 4D dose is important for scanning beam proton therapy for lung tumors.

PACS number: 87.55.D

## I. INTRODUCTION

Scanning beam proton therapy may deliver a lower dose to normal tissue than does passive scatter proton therapy (PSPT) or intensity‐modulated (photon) radiation therapy (IMRT).^(1)^ In a previous investigation,^(2)^ we developed an effective and practical method for proton therapy planning for lung tumors using internal gross tumor volume (IGTV) override, a proper smearing margin, and planning on time‐averaged computed tomography (CT; average replacement of the internal gross tumor volume or AVE_RIGTV plan) for PSPT. However, a major concern in scanning beam proton therapy for lung cancer is the dose uncertainty caused by internal organ motion resulting from breathing, which may lead to a dose distribution that differs drastically from the planned distribution. This uncertainty might be larger than PSPT in scanning beam proton therapy because of the lack of smearing margin.^(3)^ Researchers have shown that this dose uncertainty consists of two components: 1) the systematic difference between the conventionally calculated dose using a three‐dimensional (3D) CT dataset (3D dose) and the four‐dimensional (4D) accumulated dose, which is the time‐weighted average of the dose calculated in all respiratory phases (4D dose); and 2) a random component resulting from the difference between dynamically delivered and the stationary‐calculated dose.^4^, ^5^, ^6^, ^7^, ^8^ The systematic component is sometimes referred to as motion blurring (of the dose), whereas the random component is often referred to as the interplay effect; however, the definitions were previously ambiguous. Also, researchers showed that, whereas the random component can be controlled or even eliminated with the use of change delivery parameters such as repainting or elongating the treatment delivery time,^4^, ^8^, ^9^ the systematic component can only be eliminated using the 4D dose, which is an unbiased estimator of the delivered dose. Therefore, before applying the AVE_RIGTV planning technique to scanning beam proton therapy, investigating the systematic error emerging from the dose calculation performed using a 3D image set is necessary. In the study described herein, we examined the systematic error using the 3D dose, instead of the 4D dose, for scanning beam proton therapy in the treatment planning stage with dose‐volume histogram (DVH)‐ and voxel‐based techniques, and investigated the relationship between the error and lung tumor motion.

## II. MATERIALS AND METHODS

### A. Patients and plans

Ten patients with stage II‐III lung cancer treated with IMRT or PSPT at UT MD Anderson Cancer Center from March 2010 to June 2012 were selected for this retrospective study. The patients were selected for a spread of tumor motion and volume. Each patient's treatment parameters are listed in Table 1

All patients underwent 4D CT simulation^(10)^ using a T‐bar handgrip, wing boards, and a vacuum immobilization device (Vac‐Loc; CIVCO Medical Solutions, Kalona, IA) with GE LightSpeed 16 slice CT scanner (GE Healthcare, Waukesha, WI). Scans covering the entire lung volume, thorax, and upper abdomen were taken with a 2.5 mm slice spacing. The patients' respiratory phases were determined using an infrared reflecting marker and camera system (Real‐time Position Management System; Varian Medical Systems, Palo Alto, CA) and image bins corresponding to 10 equal phases, namely, T0–T90, with T0 defined as the full inhalation phase and T50 as the full exhalation phase. Each 4D CT dataset, therefore, consisted of ten 3D image sets corresponding to the 10 respiratory phases, with maximum intensity projection and averaged datasets calculated using the ten 3D image sets for treatment planning.

**Table 1:** Treatment parameters for the ten study patients with lung cancer

*Patient*	*Center of GTV Motion (mm)*	*GTV (cc)*	*CTV (cc)*	*Number of Beams*
1	9	64.4	242.4	2
2	9	70.7	196.3	3
3	17	42.8	158.9	2
4	7	80.4	299.8	3
5	5	46.6	166.9	3
6	8	165.5	539.6	2
7	10	110.3	395.1	3
8	8	73.1	203.3	3
9	12	51.9	289.5	3
10	8	46.6	191.6	3

Gross tumor volumes (GTVs) were contoured by the treating radiation oncologist using the individual phase (e.g., T50 (GTV_T50)) CT datasets. The IGTV was generated using either a union of all GTV respiratory phases or an outline of the maximum intensity CT data. The internal target volume (ITV) was defined as an 8 mm isotropic expansion of the IGTV, and the planning target volume (PTV) was defined as an expansion of the ITV by 5 mm. A clinical target volume (CTV) and PTV were generated from GTV with the same expansions on individual phases (e.g., GTV_T50, CTV_T50, and PTV_T50, respectively) for plan evaluation purposes. All normal structure contours were defined on time‐averaged CT. A PSPT plan with two or three fields was generated using the methodology that we described previously^(2)^ and reviewed by the treating physician for each patient. Single‐field optimized (SFO) and multifield optimized (MFO) scanning beam proton therapy plans^(11)^ were then generated using the Eclipse treatment planning system (version 8.9; Varian Medical Systems) at the beam angles matching the dose distribution and DVH parameters of the PSPT plan. Specifically, an averaged 4D CT set with IGTV density override (i.e., assignment of maximum CT Hounsfield unit number from individual respiratory phases)^(2)^ was used for all treatment plans.

### B. Dose calculation

A deformable registration technique was required to facilitate dose comparison and summation of different respiratory phases. To that end, an in‐house software program based on an accelerated demons algorithm was used.^(12)^ For each plan, the dose calculated on the averaged CT dataset without IGTV override was referred to as the nominal dose. Also, 3D doses were recalculated in each of the 10 phases of the simulation 4DCT using the same plan. The resulting ten dose distributions were deformed to the T50 phase for the 4D CT image set, and the 4D dose was calculated as the time‐weighted average of these ten doses. The T50 phase was used because the lung at exhalation represented the most stable and repeatable phase.^(13)^

### C. Dose analysis

As described above, T50‐phase images were used as reference image sets for planning evaluation with individual phase doses and the 4D dose. DVHs were calculated using volumes defined on T50, noting that the DVH calculated for the predeformation dose for the predeformation contour (GTV_T0) should be identical to the DVH calculated for the postdeformation dose for the postdeformation contour (GTV_T50), provided the volume of the contour did not change. In other words, the DVH was invariant during dose deformation. However, the nominal dose was calculated on averaged CT dataset. Because the target volumes (IGTVs) differed from the 4D dose (GTV_T50), deformation of the nominal dose and the target volume contours from the averaged CT to the T50 CT, or vice versa, may not have been reliable. Therefore, whereas voxel‐based comparison of the individual phase doses and 4D dose was feasible, the same comparison may not be ideal for the nominal dose and 4D dose. Therefore, an area under the curve (AUC) DVH–based method^14^, ^15^ of quantifying the difference between different dose distributions was performed.

**Figure 1:**
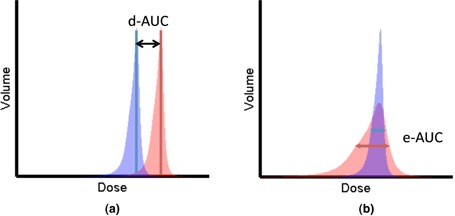
Parameterization of DVHs: (a) dAUC of two differential DVHs, (b) eAUC of two differential DVHs.

As shown in Fig. 1, two differential DVHs were calculated using different plans or images for the same organ. The difference between the mean positions of two shaded regions on the differential DVHs (dAUC, Fig. 1(a)) represented the difference in integral/mean dose received by the organ, which is equivalent to the difference in AUC for cumulative DVH. Similarly, the eAUC (Fig. 1(b)) represented the difference in dose variation within the organ between the two dose distributions.

(1) $$\begin{array}{l} e{\text{AUC}}_{V}(A,B)={\left(\sum_{D}{\left({\text{DVH}}_{V}(A)-{\text{DVH}}_{V}(B)-d{\text{AUC}}_{V}(A,B)\right)}^{2}\right)}^{1/2}\\ e{\text{AUC}}_{V}(A,B)=\sum_{D}{\text{DVH}}_{V}(A)-\sum_{D}{\text{DVH}}_{V}(B) \end{array}$$

The consistency of the pre‐ and postdeformation DVHs could easily be validated compared to the validation of deformation of dose values for the individual voxels, while it also provided extra information on the dose deformation compared to the contour deformation.

The validity of the dAUC and eAUC in the context of the 4D dose calculations was verified by correlating these two parameters with the mean and standard deviation of the difference between the doses at individual phases and the 4D dose for different volumes.

(2) $$\begin{array}{l} \mu_{V}(A,B)=\frac{1}{V}\sum_{V}D(A)-D(B)\\ \sigma_{V}(A,B)=\frac{1}{V}{\left(\sum_{V}{\left(D(A)-D(B)-\mu_{V}(A,B)\right)}^{2}\right)}^{1/2} \end{array}$$

Where μ is the mean dose difference between dose distribution A and B, while σ is the standard deviation of the dose difference. The dAUC and eAUC were then used to quantify the difference between the 3D nominal dose and the 4D dose.

## III. RESULTS

**Figure 2:**
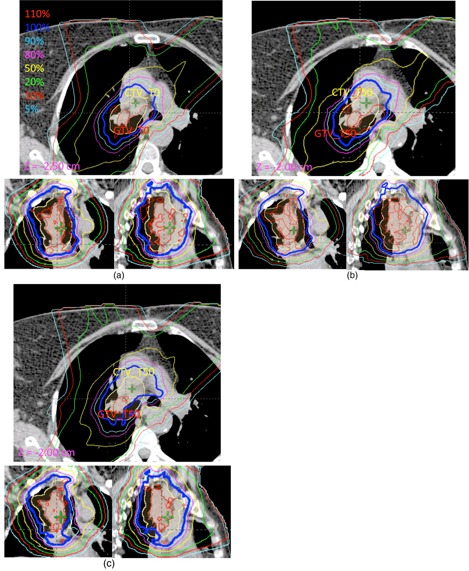
CT images showing dose deformation and 4D dose calculation for (a) one slice of a predeformed T0 dose at T0 of the 4D CT (Z = ‐2.5 cm), (b) same slice of postdeformed T0 dose at T50 of the CT (Z = ‐2.0 cm), and (c) same slice of T50 dose at T50 of the CT (Z = ‐2.0 cm).

**Figure 3:**
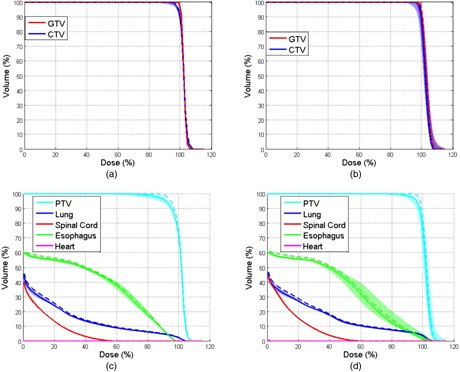
DVHs of the nominal and 4D doses in a lung cancer patient: (a) DVHs for the GTV and CTV for the SFO plan; (b) DVHs for the GTV and CTV for the MFO plan; (c) DVHs for the PTV and OARs (lung, spinal cord, heart, and esophagus) for the SFO plan; (d) DVHs for the PTV and OARs for the MFO plan.

Figures 2 and 3 show the dose deformation and 4D dose calculation for one of the studied patients. Specifically, Fig. 2(a) shows the dose distribution of the MFO plan calculated on T0 or the inhale phase of the 4D CT, while 2(c) shows the dose calculated on T50 or the exhale phase of the 4D CT. Notice the dose difference between T0 and T50 due to tumor motion indicated by the arrows. Figure 2(b) shows the dose calculated on T0 (Fig. 2(a)) deformed on to T50. The dose distribution at the same tumor location in this case is identical to Fig 2(a), as expected. Of note is that the patient coordinates differed (Z = 2.5 cm at T0 versus Z = 2.0 cm at T50) because of tumor motion. We also verified that the DVHs calculated on the predeformation doses on the predeformation contours were identical to the DVHs calculated on the postdeformation doses on the postdeformation contours.

Figure 3 shows the DVHs for the GTV, CTV, and PTV and the organs at risk (OARs; lung, spinal cord, heart, and esophagus) for the SFO (Figs. 3(a) and (c)) and MFO (Figs. 3(b) and (d)) plans. The color‐filled bands represent the ranges of DVHs calculated on different phases of the 4D CT dataset. The solid line indicates the DVH for the 4D dose calculated at T50, and the dashed line indicates the nominal dose calculated on the averaged CT. The difference between the two DVHs represents the systematic error between the 3D doses and the 4D dose.

**Figure 4:**
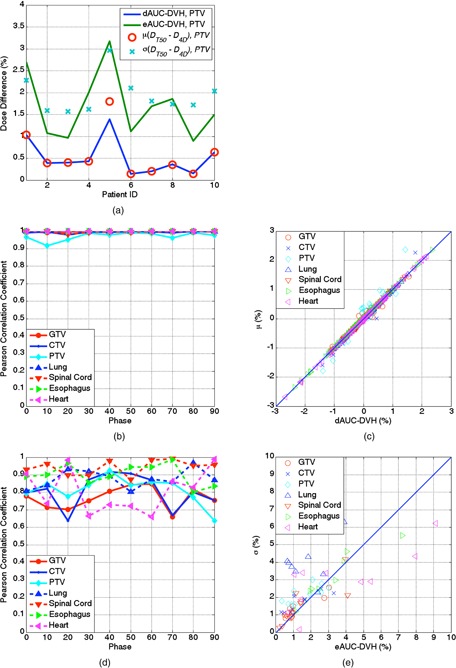
Correlation between the DVH and voxel‐based metrics for all patients: (a) plot of the dAUC, dAUC, and mean and standard deviation of the dose difference for all voxels in the PTV between $D_{T50}$ and $D_{4D}$ in the ten study patients; (b) correlation between the dAUC and mean dose difference at different respiratory phases; (c) linear fit of the dAUC and mean dose difference for all patients, structures, and phases; (d) correlation between the eAUC and standard deviation of the mean dose difference at different respiratory phases; (e) linear fit of the eAUC and standard deviation of the dose difference for all patients, structures, and phases.

Analyses of dAUC, eAUC, and voxel‐based evaluation of the PTV for different patients between T50 and 4D doses are shown in Fig. 4(a). The red circles and cyan crosses represent the mean and standard deviation of the mean ${\text{DT}}_{50}\;‐\;D_{4D}$, respectively, for voxels in the PTV for different patients, and the blue and green lines represent the dAUC(${\text{DT}}_{50}$, $D_{4D}$) and eAUC(${\text{DT}}_{50}$, $D_{4D}$), respectively. Fig. 4(b) shows the Pearson correlation coefficients for the dAUC($D_{3D}$, $D_{4D}$) and $\mu \left(D_{3D}\;‐\;D_{4D}\right)$ of the GTV, CTV, and PTV at different phases (solid lines) and for the lung, spinal cord, esophagus, and heart (dashed lines; p < 0.05 in all cases). Figure 4(c) shows the linear fit with all MFO data points (all patients, structures, and phases) between dAUC$\left(D_{3D},\;D_{4D}\right)$ and $\mu \left(D_{3D}\;‐\;D_{4D}\right)$; the overall correlation coefficient was 0.996(p < 0.01). Similarly, Fig. 4(d) shows the Pearson correlation coefficients between $\text{eAUC}\left(D_{3D},\;D_{4D}\right)$ and $\sigma \left(D_{3D}\;-\;D_{4D}\right)$, and Fig. 4(e) shows the linear fit with all MFO data points, with an overall correlation coefficient of 0.723 (p < 0.05). These results indicated that dAUC and eAUC are good metrics for quantification of the differences among dose distributions.

**Figure 5:**
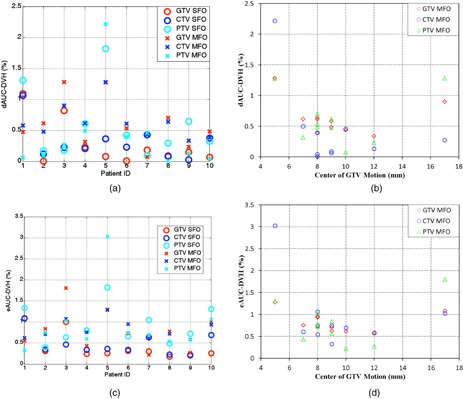
Patient‐specific evaluation of the impact of tumor motion on dose: (a) the dAUC between the nominal and 4D doses in the ten studied patients for the SFO and MFO plans; (b) the dAUC between the nominal and 4D doses as a function of tumor motion; (c) the eAUC between the nominal and 4D doses in the ten studied patients for the SFO and MFO plans; (d) the eAUC between the nominal and 4D doses as a function of tumor motion.

Figures 5(a) and 5(c) show the $\text{dAUC}\left(D_{\text{no}\min\text{al}}\;‐\;D_{4D}\right)$ and $\text{eAUC}\left(D_{\text{no}\min\text{al}}\;‐\;D_{4D}\right)$ for the GTV, CTV, and PTV with the SFO and MFO plans, respectively, and Figs. 5(b) and 5(d) show these two quantities as functions of tumor motion. The dAUC between the nominal and 4D doses, which represents the mean dose error from the 3D dose calculation, was less than 1.5% in the GTV and CTV for all patients, but was greater than 2% in the PTV for one patient. Similarly, the eAUC, which represents the standard deviation of the mean dose error, was less than 2% for all but one patient. The maximum point dose error was greater than 6% in the GTV and greater than 10% in the CTV and PTV. We found no significant differences between the dose error with the MFO and SFO plans for the GTV, CTV, and PTV according to Student's *t*‐test results.

For comparison purpose, we also repeated the calculation for the PSPT plans. Similar results were found where dAUC between the nominal and 4D doses was less than 1% in the GTV for all patients, and less than 2% for all patients in the CTV and PTV. There were statistical significant difference between PSPT and SFO (MFO) for dAUC of GTV (p < 0.05), but no significant differences were found for other quantities with Student's *t*‐test.

## IV. DISCUSSION

In this study, we applied AVE_RIGTV planning, which was developed for PSPT, to scanning beam proton therapy. Our purpose was to evaluate the systematic dose error resulting from using the nominal 3D dose instead of the 4D accumulated doses for lung cancer patients. Our results demonstrated that the systematic dose error was highly patient‐specific, but not dependent on the planning technique (SFO or MFO). The maximum point dose errors were greater than 6% for the GTV and greater than 10% for the CTV and PTV. However, the mean and standard deviation of the dose error for the GTV, CTV, and PTV was less than 1.5% for all but one patient. Although GTV coverage was maintained on all phases with IGTV override, CTV and PTV coverage could be degraded in some phases, as shown in Fig. 2. The magnitude of tumor motion was not a predictor of dose uncertainty, as the proton dose distribution was affected not only by tumor motion but also by tissue‐density change in the beam path. Overall, we found the AVE_RIGTV planning technique to be effective for scanning beam proton therapy planning of lung cancer before 4D planning and dose calculation become readily available. A point of emphasis is that patient‐specific evaluation is important and 4D dose calculations are recommended, whereas evaluation of the T0 and T50 dose should be kept as a minimum.

Previous study showed that smearing margin for setup uncertainty also played a role in taking account of target motion.^(2)^ While there was no smearing margin in scanning beam proton therapy, the lateral falloff of the spot, or the spot size, effectively serve as smearing margin. For our system with relative large spot size, our results showed that there were no statistical difference between scanning and passive scattering proton therapy for CTV and PTV. More in‐depth investigation will be necessary to fully understand the effect of spot size and spacing. The relationship between robustness and motion uncertainty, along with the investigation of optimal planning margins, will be presented in a different study.

DVHs are invariant for dose deformation given that the organ volume does not change. Therefore, dAUC and eAUC are good metrics for quantifying the variations among different doses on different patient images. Our results demonstrated that dAUC is a good predictor of the mean dose error, and that eAUC is a good predictor of the standard deviation of the dose error between 3D and 4D doses for all structures and patients. These predictors may be useful not only for 4D dose calculation, but also for adaptive planning throughout the treatment.

## V. CONCLUSIONS

In this study, we evaluated the systematic dose error resulting from 3D dose calculation for scanning beam proton therapy. The dose error in 3D dose calculation varies from patient to patient and does not correlate with lung tumor motion. Therefore, patient‐specific evaluation of the 4D dose is important for scanning beam proton therapy for lung tumors. Before 4D planning and dose calculation become readily available, AVE_RIGTV planning technique is effective for scanning beam proton therapy planning of lung cancer.

## ACKNOWLEDGMENTS

We thank Christine Wogan Patterson from the Department of Scientific Publications at MD Anderson Cancer Center for editorial review of this manuscript. MD Anderson Cancer Center is supported by the National Institutes of Health through grant CA16672.
